# Color Evaluation of Tinted Sunscreens on Diverse Skin Tones

**DOI:** 10.1111/jocd.71240

**Published:** 2026-10-11

**Authors:** Hawasatu Dumbuya, Mariana Mitie Yamamoto, Laure Munoz, Alix Danoy, Tania Katherine Hernandez, Loic Colomb, Zoe Diana Draelos

**Affiliations:** ^1^ La Roche‐Posay Laboratoire Dermatologique L'Oréal USA New York New York USA; ^2^ QIMA Life Sciences, QIMA Newtone SAS Lyon France; ^3^ Dermatology Consulting Services, PLLC High Point North Carolina USA

**Keywords:** color matching, patient sunscreen compliance, skin of color, tinted sunscreen, visible light

## Abstract

**Background:**

Topical formulations incorporating visible light (VL)‐attenuating pigments, specifically iron oxides (FeO) alongside broad‐spectrum sunscreens, protect against VL‐induced hyperpigmentation and aid in managing pigmentary disorders. However, the market suffers from limited shade ranges that often exclude individuals with darker skin tones; even self‐proclaimed “universal” shades frequently leave an aesthetically unacceptable “ashy” or grayish cast.

**Objective:**

Since the cosmetic elegance of sunscreens is critical for patient compliance, this study aims to evaluate the color matching and acceptability of seven commercially available tinted sunscreens in a diverse cohort of FST III–VI individuals.

**Methods:**

The design consists of a single‐center, double‐blind, randomized, split‐face study, including in a diverse cohort of FST III–VI individuals (*n* = 40). Color matching and acceptability of seven commercially available tinted sunscreens (tailored mineral, universal mineral, and hybrid formulations) were evaluated using both investigator and subject questionnaires, plus clinical imaging and analyses.

**Results:**

During baseline swatch testing, participants with FST V–VI unanimously rejected the “universal” inorganic sunscreen. Furthermore, investigator and subject questionnaires, plus image analyses revealed that while tailored tinted formulations (inorganic or hybrid) achieved high‐to‐moderate satisfaction for FST III–IV phototypes, scores dropped notably for individuals with higher FST and deeper complexions. Our data show that skin evenness and blendability are just as vital to product adoption as preventing visible residue. Collectively, our findings demonstrate that optimizing FeO variants and UV filter configurations in sunscreen formulations is critical to achieving consumer satisfaction across diverse populations.

**Conclusion:**

We hope this research clarifies the key factors driving successful color matching and satisfaction in individuals with SOC, thereby optimizing sunscreen compliance and clinical outcomes for patients with darker skin tones.

## 1 Introduction

Ultraviolet (UV) radiation is well‐established in the pathogenesis of cutaneous damage; however, nearly 45% of the sunlight that reaches the earth comprises visible light radiation (VL, 400–700 nm) [1, 2]. In particular, the high‐energy visible light portion (HEVL, 415–455 nm) has emerged as a profound driver of persistent dyschromia, particularly in individuals with Skin of Color (SOC) of Fitzpatrick Skin Types (FST) III through VI [3, 4, 5, 6].

Despite the clear impact of VL on pigmentary disorders, traditional broad‐spectrum non‐tinted sunscreens relying solely on organic and inorganic UV filters are virtually transparent to the visible light spectrum, offering negligible protection against VL‐induced hyperpigmentation [7, 8, 9]. To bridge this photoprotective gap, topical formulations must incorporate visible light‐attenuating pigments, specifically varying ratios of yellow, red, and black iron oxides (FeO) [10, 11, 12]. Clinical studies demonstrate that the addition of FeO to broad‐spectrum sunscreens significantly blocks HEVL transmittance and is mandatory for effective long‐term management and relapse prevention of melasma [7, 8, 13]. However, the daily use of FeO‐containing sunscreens (or tinted sunscreens) is frequently undermined by a critical real‐world barrier: patient compliance [14]. The lack of suitable color options for diverse skin tones often results in formulations with poor cosmetic elegance and color integration [15]. On deeper skin tones, these products frequently leave an aesthetically unacceptable “ashy,” grayish, or white cast [15, 16, 17, 18].

One of the challenges with tinted sunscreens is blendability that allows the sunscreen to function optimally by creating a thin even film and meeting the consumer's desire for optimal aesthetics. To address these aesthetic challenges in individuals with SOC, the cosmetic industry has popularized “universal” tinted sunscreens, marketed to blend seamlessly across all human skin tones [15, 16]. However, recent clinical evaluations challenge the validity of this claim [15, 16, 17, 18]. A 2024 study utilizing colorimetry demonstrated that “universal” tinted sunscreens fail to provide adequate color matching for higher Fitzpatrick skin phototypes, leaving cosmetically unacceptable white cast and dark residues, particularly in FST V and VI [16]. Given the importance of cosmetic elegance for patients' sunscreen compliance, this study aims to evaluate the color matching, and key aspects of product acceptability of seven different tinted sunscreens (tailored mineral, universal mineral, and hybrid formulations) in diverse racial/ethnic individuals with skin phototypes FST III–VI, using investigator and subject questionnaires, plus image analyses.

## 2 Materials and Methods

### 2.1 Study Design and Demographic

A single‐center, double‐blind, randomized, split‐face clinical trial was conducted to evaluate the cosmetic blendability and color adaptability of seven commercial tinted sunscreens. The study adhered to the Good Clinical Practice (GCP) guidelines and the principles outlined in the Declaration of Helsinki. The study protocol and all associated materials were reviewed and approved by an independent Institutional Review Board [Allendale Institutional Review Board (AIRB), Old Lyme, CT] prior to study initiation. Written informed consent and photography release forms were obtained from all subjects prior to enrollment and any study‐related procedures.

A total of forty (40) healthy male and female subjects, aged 18 years and older, were enrolled in the study, comprising exactly ten (10) subjects each for FST III, IV, V, and VI. The cohort encompassed a highly diverse demographic, including subjects of African American (31), Hispanic/Latin (7), and Asian (2) descent. Participants presented with self‐perceived dyschromia, uneven skin tone, or dull skin. Key exclusion criteria included pregnancy or lactation, active dermatologic conditions on the face, recent excessive UV exposure or sunburn, and the use of systemic or topical corticosteroids within one week prior to the study. Subjects were required to arrive at the clinic with a clean face, devoid of any prior cosmetic applications.

### 2.2 Investigational Products

The study evaluated a total of seven (7) commercially available tinted sunscreens: The investigational group comprised four (4) tailored tinted mineral sunscreens from La Roche‐Posay US, representing a graduated shade range: original, medium, medium‐deep, and deep. The comparator group consisted of three (3) commercial products: one (1) mineral‐tinted sunscreen marketed as a “universal” shade and two (2) hybrid (a combination of organic and inorganic UV filters systems) tinted sunscreens available in light and dark shades. The study employed a double‐blind design in which all tinted sunscreen products were masked to remove recognizable brand or packaging elements. This ensured that recognizable product attributes did not influence application or assessments for clinical participants, technicians, and investigator dermatologist.

To simulate real‐world consumer behavior, a baseline swatch test was performed on the clean skin of the lower cheek and jawline, where each subject self‐selected one preferred shade from the tailored mineral shade range (original, medium, medium‐deep, and deep), as well as one preferred formulation from the comparator group (choosing either the ‘universal’ mineral shade or one of the two hybrid sunscreens), as illustrated in Figure 1. Following subjects' tinted sunscreens selections, application of products was performed on each assigned side of the face following a randomization list by a trained clinical technician at 1 mg/cm^2^.

**FIGURE 1:**
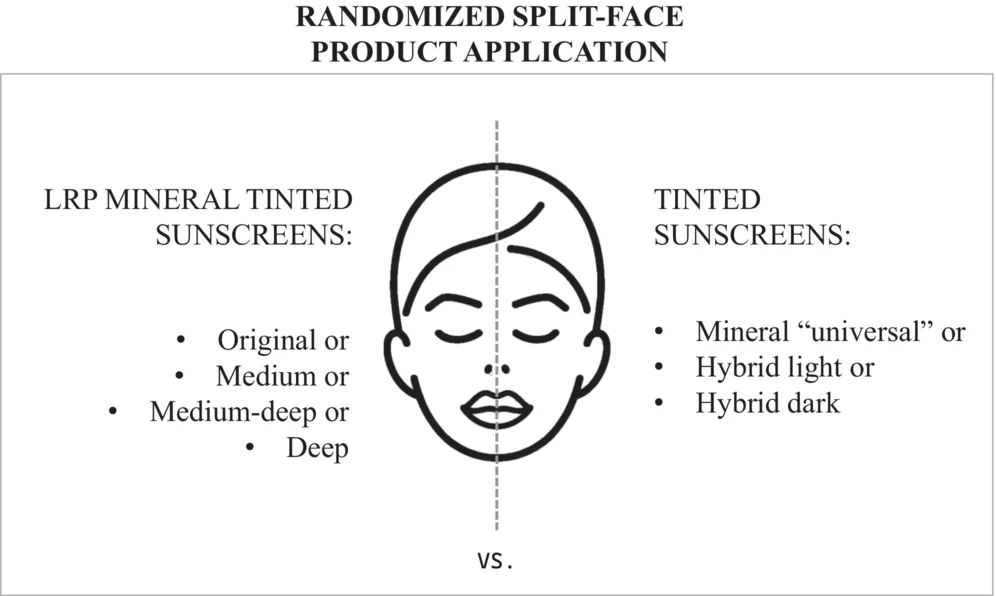
Schematic representation of randomized product application. Following a baseline jawline swatch test, study participants self‐selected their preferred tinted sunscreen shades. A trained technician then performed randomized, split‐face applications of the products at a controlled dose of 1 mg/cm^2^.

### 2.3 Investigator Questionnaires

A blinded expert dermatologist evaluated the formulations 15 min post‐product application on patients with questionnaires, assessing the following parameters: color match, absence of an ashy/white cast, skin evenness, overall complexion, and recommendation intent. The use of a single blinded dermatologist was an intentional methodological choice designed to reflect real‐world clinical practice. Because sunscreen counseling typically occurs in a one‐on‐one setting between a single clinician and a patient, this approach allowed us to authentically capture the nuances of color‐matching perception and preference alignment between the healthcare provider and the patient, providing valuable clinical insights, particularly for individuals with skin of color.

### 2.4 Subject Self‐Assessment Questionnaires (SAQ)

Subjects self‐evaluated the products following application with questionnaires on perceived color integration, absence of white residue, skin evenness, complexion improvement, and product replacement intent.

All investigator and subject questionnaires responses were recorded using a standardized 9‐point Likert scale, where 9 = Completely Agree, 7 = Somewhat Agree, 5 = Neither Agree nor Disagree, 3 = Somewhat Disagree, and 1 = Completely Disagree.

Questionnaires results were analyzed utilizing two distinct methodologies:

1. Top‐Box Scoring (bar charts): To establish a threshold of excellence for cosmetic acceptability, individual aesthetic parameters (e.g., color match, absence of white cast) were evaluated based on the percentage of subjects awarding a “top‐box” score (defined exclusively as an 8 or 9).
2. Multidimensional Mean Analysis (radar charts): To assess holistic formulation performance and overall cosmetic elegance across multiple sensory domains simultaneously, the mean scores for each parameter were calculated and plotted on radar (spider) charts. This allowed for a visual, multidimensional comparison of color integration aspects, cosmetic elegance, and product recommendation/replacement intent across different skin phototypes.

### 2.5 Statistical Methods

Descriptive statistics were calculated for each self‐assessed and investigator‐graded endpoint by group. These included means, standard deviations, and the proportion of subjects scoring within the top categories (defined as 8 or 9 on a 9‐point Likert scale). Results are presented graphically as radar charts and bar graphs. Statistical comparisons between groups were conducted using Wilcoxon rank‐sum and Fisher's exact tests on an exploratory basis to inform interpretation of observed patterns. Given the limited power of these tests due to the small sample sizes and the exploratory nature of the analysis, results are presented descriptively rather than through hypothesis testing, and *p*‐values are not reported.

### 2.6 2D Imaging Colorimetric Analyses

Objective evaluation of skin color alteration was measured at baseline (bare skin, no product) and 15 min post‐ sunscreen application using image analysis. Standardized, high‐resolution photographs of all subjects (full face) were captured using the VISIA‐CR system under standard, cross‐polarized, parallel‐polarized, and UV lighting modalities. QIMA Newtone 2D image analysis software was utilized. Standardized digital masks (regions of interest) were applied to the images to extract pixel‐by‐pixel colorimetric data (Figure 6A). The absolute color deviation induced by the formulations was calculated using the CIELAB parameters: lightness (L*), redness (a*), and yellowness (b*) color difference to quantify:

1. The ΔE76 parameter, which quantifies the total color shift before and after tinted sunscreen application. The higher the value, the greater the difference in color. It was calculated with the following formula:

$$\mathrm{\Delta E}76=\sqrt{{\left(\Delta L^{*}\right)}^{2}+{\left(\Delta a^{*}\right)}^{2}+{\left(\Delta b^{*}\right)}^{2}}$$

2 The Individual Typologic Angle (ITA°), which characterizes skin typology or pigmentation. The lower the value, the darker the skin pigmentation or complexion. It was calculated with the following formula:

$$\begin{array}{c} \mathrm{ITA}{}^{\circ}=\left[\mathrm{Arc}\;\text{Tangent}\;\left(\left(L^{*}-50\right)/b^{*}\right)\right]\;180/\pi \end{array}$$

## 3 Results

A total of forty (40) subjects completed the study using a randomized, split‐face protocol, resulting in 80 total product applications. Table 1 shows the specific tinted sunscreen formulations chosen by the participants following a baseline swatch test to determine the best aesthetic match. These selections are stratified by racial/ethnic background (African American, Asian, and Hispanic; Table 1A) and Fitzpatrick Skin Type (FST; Table 1B). As expected, FST III–IV subjects selected lighter tinted sunscreen, while FST V–VI subjects chose darker tinted sunscreens. As indicated in Table 1A,B, there was a wide selection frequency across FST III through VI, underscoring the diverse shade matching requirement for populations with SOC of different ethnicities. Notably, the universal mineral tinted sunscreen was not selected by any subjects in the FST V–VI cohort due to immediate aesthetic unsuitability.

**TABLE 1:** Clinical participants demographics & tinted sunscreen selection. Forty subjects completed the study using a randomized, split‐face protocol, resulting in 80 total product applications. (A) Highlights self‐reported racial/ethnicity with product selection, while (B) displays selection frequency across Fitzpatrick skin phototype III–VI. The seven evaluated products consisted of four La Roche‐Posay (LRP) US mineral sunscreens (original to deep shades) and three comparator products: One ‘universal’ mineral sunscreen and two hybrid (organic/inorganic) sunscreens in light and dark shades.

(A)				
	Ethnicity			Total
Product	AA	AS	H	
LRP original	1	2	3	6
LRP medium	10	0	2	12
LRP medium‐deep	15	0	2	17
LRP deep	5	0	0	5
Mineral universal	3	0	2	5
Hybrid light	9	2	4	15
Hybrid dark	19	0	1	20
Total	62	4	14	80

(B)					
	Fitzpatrick skin phototype				Total
Product	III	IV	V	VI	
LRP original	6	0	0	0	6
LRP medium	2	5	3	2	12
LRP medium‐deep	2	4	7	4	17
LRP deep	0	1	0	4	5
Mineral universal	2	3	0	0	5
Hybrid light	7	4	2	2	15
Hybrid dark	1	3	8	8	20
Total	20	20	20	20	80

*Note:* 10 subjects per Fitzpatrick skin phototype.

Abbreviations: AA = African American, AS = Asian, H = Hispanic.

### 3.1 Investigator Assessments

Investigator‐graded aesthetic performance of two (2) parameters (color integration and absence of white cast) is shown in Figure 2. Results compare the four (4) tailored mineral tinted formulations (original, medium, medium‐deep, and deep) with three (3) commercial products: one (1) mineral tinted sunscreen marketed as a “universal” shade, and two (2) hybrid (a combination of organic and inorganic UV filter systems) tinted sunscreens available in light and dark shades. Data is stratified by skin phototype (FST III–IV vs. V–VI) for the absence of a white/ashy cast (Figure 2A) and color match (Figure 2B) for each individual sunscreen. Bar graphs illustrate the percentage of subjects achieving top clinical scores (defined as 8 or 9 on a 9‐point Likert scale), as evaluated by an expert dermatologist. All tinted sunscreen formulations achieved high and similar satisfaction scores (≥ 80%) regarding the absence of white cast in FST III–IV cohort (Figure 2A). For the cohort with FST V–VI, investigators reported moderate scores for leaving no white cast across nearly all formulas (≥ 75%). The exception was the tailored mineral medium shade (40%); as anticipated, its lighter tint was more visible on darker skin tones (Figure 2A). Regarding color matching, investigators reported higher scores (≥ 83%) in the FST III–IV cohort for three of the four tailored mineral shades—original, medium‐deep, and deep—as well as the hybrid light formulation (Figure 2B). In contrast, within the FST V–VI subgroup, only the hybrid dark formulation achieved a high satisfaction score above 90%, followed by tailored mineral deep and hybrid light (both at 75%), and medium‐deep (72%). These findings reveal a divergence between color‐matching efficacy and white‐cast perception, underscoring the complex aesthetic requirements of diverse skin tones.

**FIGURE 2:**
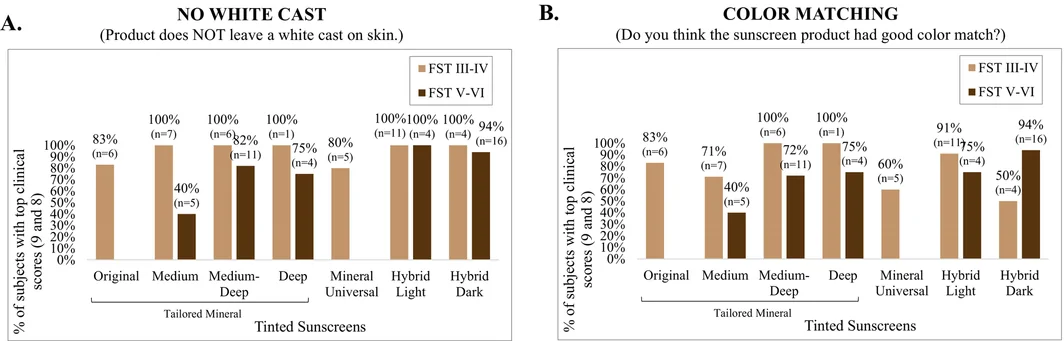
Dermatologist assessment of white cast and color match across tinted sunscreens by phototype. Graphs show the percentage of subjects achieving top‐box scores (8–9) on a 9‐point Likert scale as evaluated by board‐certified dermatologist 15 min post‐product application. Results are stratified by FST III–IV and V–VI for (A) absence of white cast and (B) skin color matching.

Multi‐dimensional mean analysis (radar charts), illustrating the mean dermatologist‐graded scores for five (5) cosmetic performance parameters (Color matching, no white cast, skin evenness, overall complexion, and product recommendation based on blendability), stratified by skin phototype subgroups are shown in Figure 3. Scores are mapped on a 5–9 axis (from a standard 9‐point scale) to delineate formulation differences. Figure 3A represents the lower skin phototypes (FST III–IV), and Figure 3B represents higher skin phototypes (FST V–VI). The area of each polygon represents the overall multidimensional color integration of the respective formulation. Overall aesthetic satisfaction, perceived by the investigator, was achieved for nearly all products, except for hybrid dark shade sunscreen in the FST III–IV group (Figure 3A). Perceived aesthetic performance, by investigator, was more nuanced within the FST V–VI group (Figure 3B). Both light and dark hybrid formulations demonstrated comparable high performance, while the tailored mineral shades showed a preference gradient, darker shades performing better than lighter shades. Notably, the universal mineral tinted sunscreen yielded no data for the FST V–VI cohort, as subjects universally rejected it due to immediate aesthetic unsuitability. Overall, these findings emphasize that a careful balance of UV filters and FeO pigments is essential for optimizing the perceived aesthetics of tinted formulations.

**FIGURE 3:**
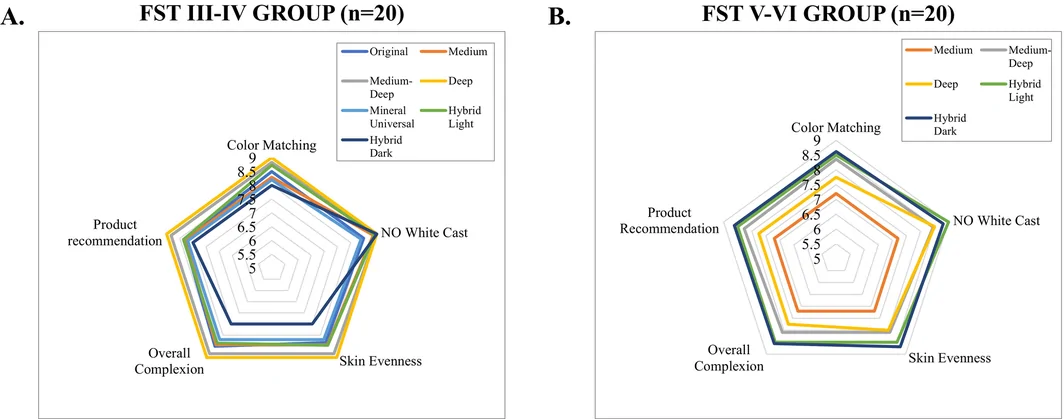
Dermatologist assessment of five cosmetic performance parameters across tinted sunscreens. Radar charts illustrate mean dermatologist‐graded scores across five parameters 15 min post‐application: Color matching, no white cast, skin evenness, overall complexion, and product recommendation (based on blendability). To clearly delineate performance, scores are mapped on a 5–9 axis (from a standard 9‐point Likert scale). Results are stratified by (A) FST III–IV and (B) FST V–VI.

### 3.2 Subject Assessments

Subjects' perception and user experience of color integration and absence of white cast are shown in Figure 4. Bar graphs illustrate the percentage of subjects awarding top satisfaction scores (8 or 9 on a 9‐point Likert scale) during self‐evaluation. Data is stratified by FST III–IV versus V–VI for the absence of a white residue (Figure 4A) and perceived skin color match (Figure 4B). While FST III–IV subjects reported consistent moderate satisfaction (≥ 75%) with the absence of white cast across all formulas, perceptions in the FST V–VI cohort were more nuanced. Specifically, satisfaction with the tailored mineral medium shade and hybrid light comparator dropped to 40% and 50%, respectively. As anticipated, these lighter tints were more visible on darker skin, whereas deeper‐tinted formulations were perceived more favorably, achieving satisfaction scores of 88% or higher (Figure 4A). In terms of skin color matching (Figure 4B), FST III–IV subjects reported moderate satisfaction (≥ 75%) for most products, apart from the tailored mineral medium‐deep and universal mineral formulations, which scored lower at 67% and 60%, respectively. Interestingly, subjects in the FST V–VI cohort rated both hybrid products (light and dark) similarly to the deepest tailored mineral shades (medium‐deep and deep). These subject‐reported trends align with the initial investigator assessments (Figures 2B and 4B), underscoring the critical divergence between skin color‐matching and the presence of white‐cast residue in achieving aesthetic satisfaction for darker skin tones.

**FIGURE 4:**
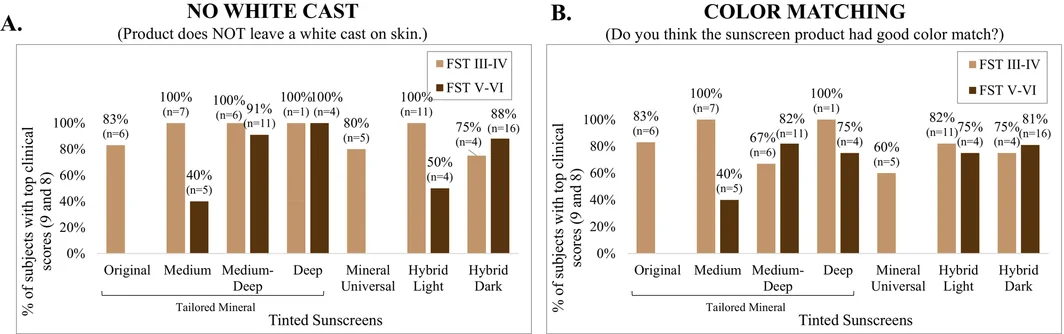
Subjective assessment of white cast and color match of tinted sunscreens by phototype. Graphs show the percentage of subjects achieving top‐box scores (8–9) on a 9‐point Likert scale as evaluated by study subjects 15 min post‐product application. Results are stratified by FST III–IV and V–VI for (A) absence of white cast and (B) skin color matching.

Subject evaluations of the same five (5) cosmetic performance parameters, previously evaluated by the investigator, also stratified by skin phototype subgroups, are shown in Figure 5. In the FST III–IV cohort, the tailored mineral medium and deep shades achieved the highest satisfaction levels for color matching and skin evenness, which correlated with a high intent for product replacement (Figure 5A). Conversely, FST V–VI subjects reported much lower satisfaction across all five evaluated cosmetic parameters for all products (Figure 5B). This broad aesthetic failure resulted in markedly lower product replacement intent, underscoring a critical barrier to photoprotection compliance in deeper skin tones. Ultimately, these findings reveal that for darker skin, tinted sunscreens' aesthetic satisfaction is driven by a complex interplay of attributes beyond the mere absence of white residue.

**FIGURE 5:**
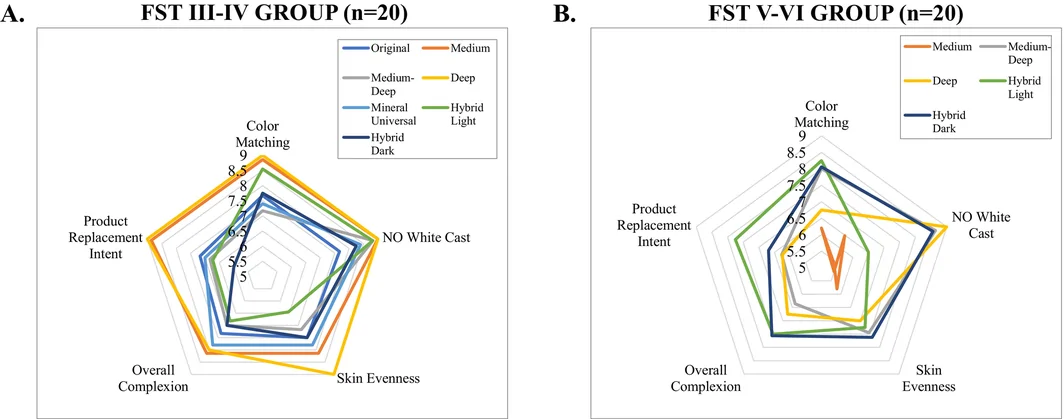
Subjective evaluation of five cosmetic performance parameters across tinted sunscreens. Radar charts illustrate subject self‐assessment mean scores across five parameters 15 min post‐application: Color matching, no white cast, skin evenness, overall complexion, and product replacement intent (based on blendability). To clearly delineate performance, scores are mapped on a 5–9 axis (from a standard 9‐point Likert scale). Results are stratified by (A) FST III–IV and (B) FST V–VI.

### 3.3 2D Clinical Image Analyses

Standardized VISIA‐CR images were digitally processed to measure absolute chromatic changes before and after product application. As illustrated by the representative methodology in Figure 6A, a standardized digital region of interest was defined on each cheek area (purple area) to extract pixel‐by‐pixel colorimetric data. This generated the mean lightness (L*), redness (a*) and yellowness (b*) values of each cheek to enable the calculation of the total color difference parameter (ΔE76), from baseline for each formulation, stratified by skin phototype; higher ΔE76 values indicate a greater mathematical deviation from the subject's natural bare skin (Figure 6B). Overall, image analyses revealed high ΔE76 values for three out of the four tailored mineral shades (original, medium, and medium‐deep) within the FST III–IV cohort. Interestingly, these instrumental color shifts were not perceived as negative skin tone changes; instead, both investigators and subjects rated them favorably (Figures 4 and 5). This suggests that high ΔE76 values reflected positive cosmetic enhancements, possibly desirable coverage and improved complexion evenness, rather than a shade mismatch. Conversely, the FST V–VI cohort exhibited lower ΔE76 values for nearly all products (except the tailored mineral medium and deep shade), indicating minimal shift from bare skin. However, these colorimetric instrumental results diverged from subject evaluations, where participants still reported dissatisfaction with color matching and complexion blendability.

**FIGURE 6:**
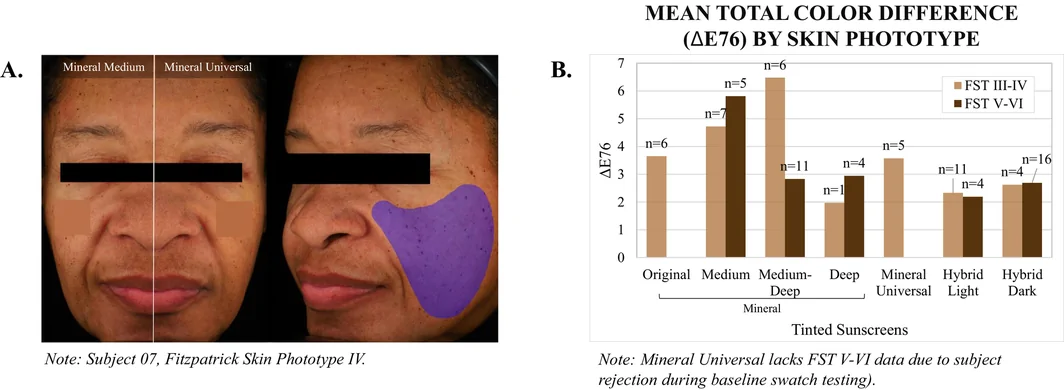
2D imaging colorimetric analyses of tinted sunscreens by phototype. Standardized VISIA‐CR images were digitally processed to quantify colorimetric changes post‐product application. As shown in (A), a standardized region of interest (ROI) on the cheek was utilized to extract pixel‐by‐pixel 2D data. (B) This analysis generated the mean total color difference (ΔE76) from baseline for each formulation, with results stratified by FST III–IV and V–VI subgroups.

Subsequently, to further characterize the colorimetric shifts induced by tinted product application and assess their impact on perceived aesthetic efficacy, we utilized image analysis to quantify changes in CIELAB parameters: lightness (L*), redness (a*), and yellowness (b*) in selected clinical participants (Figures 7 and 8). By comparing baseline bare skin to the immediate post‐application state, we sought to determine the correlation between these objective colorimetric shifts and subjective subject preference. An initial comparison was conducted between the tailored mineral deep shade and the mineral universal sunscreens to determine their respective impacts on skin color change. On the selected subject with FST IV (Figure 7A), application of the universal tinted sunscreen resulted in a measurable reduction in skin redness (Δa*, −2.27) and yellowness (Δb*, −4.04), plus a darker skin pigmentation or complexion (ΔITA°, −3.38) appearance, which led to poorer subject feedback regarding color match and white cast (Figure 7C). In contrast, tailored mineral deep shade preserved the skin redness and yellowness without major deviation; yet elicited a noticeable decrease in skin lightness (ΔL*, −1.60) and shifts toward a darker overall complexion (ΔITA°, −2.91) compared to baseline bare skin; these specific shifts were highly satisfied by same participant, who provided top ratings across all five aesthetic attributes (Figure 7C).

**FIGURE 7:**
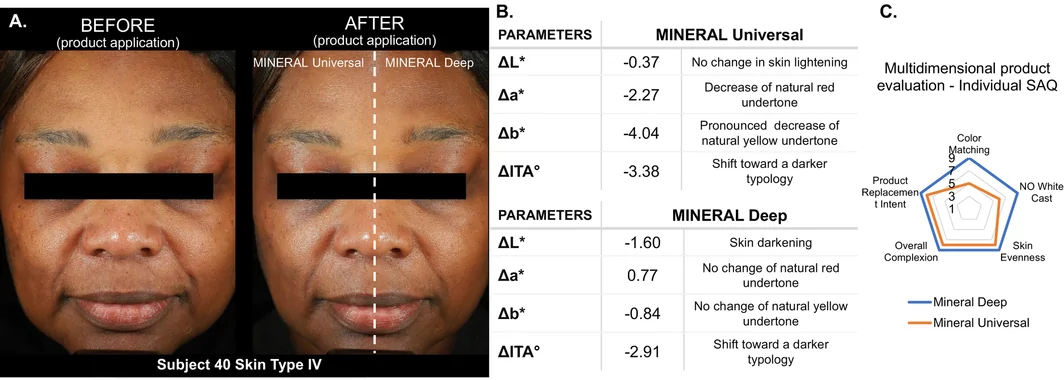
Multidimensional evaluation case study (Subject 40, FST IV) for tinted sunscreen performance. (A) Standardized VISIA‐CR split‐face photography comparing bare skin to the immediate post‐application of mineral universal (left) and tailored mineral deep (right) tinted formulations. (B) Quantitative colorimetric 2D imaging analysis detailing the specific skin color shifts (ΔL*, Δa*, Δb*, ΔITA°). (C) Radar plot of the subject's self‐assessment questionnaire (SAQ).

**FIGURE 8:**
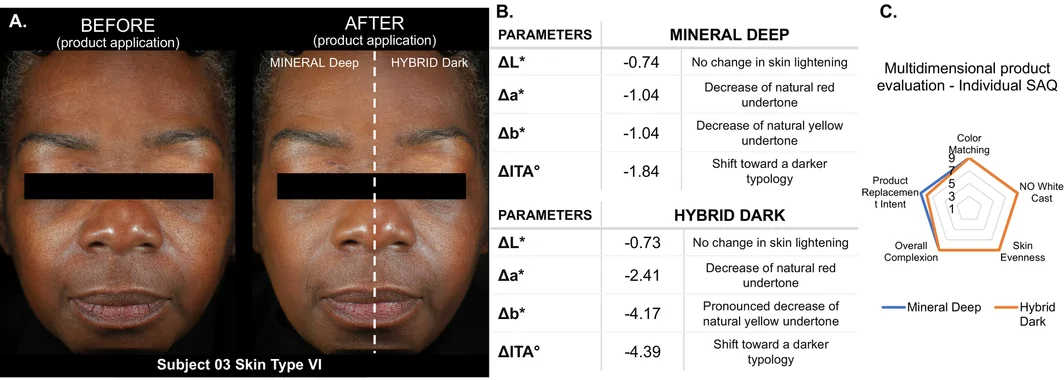
Multidimensional evaluation case study (Subject 03, FST VI) for tinted sunscreen performance. (A) Standardized VISIA‐CR split‐face photography comparing bare skin to the immediate post‐application of tailored mineral deep (left) and hybrid dark (right) tinted formulations. (B) Quantitative colorimetric analysis detailing the specific color shifts (ΔL*, Δa*, Δb*, ΔITA°). (C) Radar plot of the subject's self‐assessment questionnaire (SAQ).

Next, we compared the two deepest shades—the tailored mineral deep and the hybrid dark tinted sunscreens—to assess their respective colorimetric impacts on a subject with FST VI (Figure 8A). In this participant, the tailored mineral deep shade resulted in a comparable decrease in skin redness (Δa*) and yellowness (Δb*) (both at −1.04). This was accompanied by a subtle shift toward a deeper complexion (ΔITA°, −1.84) compared to baseline bare skin (Figure 8B). This particular subject with FST VI reported high satisfaction with these specific shifts, providing top ratings for all five aesthetic attributes, including color matching, skin evenness and overall complexion (Figure 8C). Comparatively, the hybrid dark formulation induced a reduction in redness (Δa*, −2.41) and a more pronounced decrease in yellowness (Δb*, −4.17), yielding a visible deeper complexion (ΔITA°, −4.89) relative to both bare skin and the tailored mineral deep shade. These colorimetric shifts were perceived favorably by the subject, who reported high satisfaction across all five aesthetic attributes. However, the hybrid formulation received a slightly lower score for product replacement intent. Collectively, these image analyses indicate that FeO concentrations and UV filter configurations must be optimized to drive aesthetic efficacy across a diverse range of skin tones. Certain colorimetric variations following product application should be interpreted as cosmetic benefits—including effective coverage and enhanced evenness—rather than shade mismatches. The perception of these shifts as positive enhancements remains contingent upon their chromatic alignment with the subject's baseline pigmentation and natural undertones.

## 4 Discussion

The daily application of FeO‐containing tinted sunscreens is essential for mitigating HEVL‐induced dyschromia in individuals with SOC [7, 8, 9, 14, 19, 20]. However, sunscreens benefits are intrinsically tied to individual compliance and adherence [14, 21]. Despite being excluded from FDA monograph, FeO additive ingredients in tinted sunscreens have become the most frequently recommended by dermatology practitioners for visible light protection [22]. Several guidelines have emerged to help and advise clinicians and patients on tinted sunscreen selection and usage, considering ingredients and cosmetic suitability [19, 23, 24, 25]. Torres et al. suggested that consumers should choose tinted sunscreens containing iron oxides, labeled as broad‐spectrum, and with an SPF of at least 30 [23]. Torres et al. also recommended that the tinted suncare product should also match the user's skin tone for cosmetic appeal [23]. However, several reports highlight that individuals with SOC of darker skin tone face several specific challenges when using these tinted sunscreens, such as limited shade diversity, tone incompatibility, aesthetic discomfort, higher cost, lack of ingredient transparency and practical application issues [15, 16, 17, 18, 25]. In view of these challenges, we sought to identify the aesthetic attributes (positive and negative) of different tinted suncare formulations in individuals with deeper skin tones, utilizing a randomized split‐face design, investigator and subject questionnaires, plus clinical imaging. In summary, our multidimensional study reveals that while progress has been made in mitigating the white cast of mineral sunscreens for individuals with SOC, modern formulations primarily achieve cosmetic success for FST III–IV. A persistent gap remains in addressing the specific aesthetic and undertone requirements for darker skin tones (FST V–VI), highlighting a significant area for future industry innovation.

We designed the study to simulate real‐world consumer behavior, where participants first performed a baseline swatch test on their jawline to evaluate different tinted sunscreens options. While this approach effectively mirrors natural consumer habits, it introduced a methodological limitation in uneven and occasionally small subgroup denominators. To ensure transparent data interpretation, these sample sizes are explicitly noted in each figure. Based on this test, subjects self‐selected their preferred shades for the subsequent facial application phase of the study. It was interesting to observe that during baseline swatch testing, participants of darker skin tones with FST V–VI unanimously rejected the mineral universal sunscreen (Table 1). While the non‐selection of the lightest tailored shades (original) by this cohort was anticipated, the unanimous rejection of the ‘universal’ formulation by subjects with deeper skin tones underscores the technical inadequacy of a one‐size‐fits‐all pigment strategy. These data support the clinical necessity of diversifying FeO ratios to ensure aesthetic suitability and chromatic alignment across the entire range of demographics with SOC.

Since ‘white cast’ and ‘chalky/ashy’ residues are frequently cited as negative attributes driving poor sunscreen compliance within the general public, especially in communities with SOC, we sought to explore the correlation between white cast and color matching across various tinted formulations, examining the specific influence of FeO types and concentrations plus UV filter systems in a racially and ethnically diverse cohort. Unexpectedly, the attributes of white cast and color matching did not always align across investigator and subject evaluations (Figures 2 and 4). For participants in the FST V–VI cohort, it was notable that while the deepest tailored mineral shades achieved high satisfaction for the absence of white cast (91% for medium‐deep, 100% for deep), they received lower scores for color matching at 82% and 75%, respectively (Figure 4A,B). Similarly, FST V–VI participants who self‐selected the light hybrid formulation reported only 50% satisfaction regarding white cast, despite an 83% satisfaction rate for color matching performance. Beyond color matching and white cast, the subjects' evaluations of skin evenness, overall complexion, and blendability revealed a multifaceted layer of product satisfaction. While no white cast was vital, subjects identified skin evenness, complexion enhancement, and blendability as key determinants of their satisfaction. Specifically, for the FST V–VI group, deficiencies in these areas appeared to be the primary drivers of low product adoption (Figure 5B).

Given that investigator and subject questionnaires are inherently subjective, several research groups have utilized objective colorimetry to evaluate the shade‐matching potential of marketed tinted sunscreens [16, 17, 18, 25]. Kohli et al. demonstrated that ‘universal’ tints significantly increase skin lightness post‐application across all skin types, with the magnitude of this colorimetric discrepancy correlating positively with the FST [17]. Furthermore, Gawey et al. recently identified that top‐selling ‘universal’ formulations primarily cluster within the FST III–IV range, effectively underserving the lowest FST (I–II) and highest FST (V–VI) phototypes [18]. Notably, Gawey et al. revealed a great market predominance of warm (red‐yellow) undertones over cool chromatic profiles [18]. In our study, to further investigate the drivers of aesthetic efficacy we used 2D image analysis to quantify changes in CIELAB parameters: lightness (L*), redness (a*), and yellowness (b*) in selected clinical participants with FST IV and VI. By integrating colorimetric data with subject questionnaires, we aimed to determine the impact of objective color variations on subjective aesthetic attributes and overall product acceptability. We observed that the universal tinted sunscreen induced a noticeable attenuation of natural red and yellow chromatic components in the selected subject with FST IV when compared to baseline bare skin and tailored mineral deep shade (Figure 7). Unfortunately, and as mentioned, data for deeper skin tones (FST V–VI) were unavailable, as the universal product was rejected by all participants in these categories during the initial swatching assessment, underscoring its limited aesthetic compatibility. In evaluating the two deepest shades—the tailored mineral deep and the hybrid dark formulations—distinct colorimetric trends were observed based on the subject's phototype and natural skin tone. For the selected FST VI participant, application of tailored mineral deep and the hybrid dark formulations resulted in an attenuation of red and yellow undertones, shifting the complexion toward a cooler chromatic profile; this reduction in warm undertones and overall complexion was more pronounced by the hybrid dark formulation (Figure 8). Conversely, in other FST VI subjects, these specific deeper formulations induced a pronounced yellow deposit and an upward shift in skin lightness (*data not shown*). Although our cohort includes a higher proportion of African American participants, it features a balanced distribution across Fitzpatrick skin phototypes III–VI. As detailed in our demographic tables, the standard Fitzpatrick scale has limitations when evaluating individuals of color, as it often fails to capture the distinct nuances of race, ethnicity, and underlying skin tone within a single phototype category (Table 1A,B). Consequently, our nuanced observations emphasize that undertones may play a critical, individualized role in sunscreen color matching, supporting a tailored clinical approach rather than a one‐size‐fits‐all standard.

Collectively, these findings indicate that the optimization of FeO variants and UV filter, and overall formulation configurations is critical for achieving high consumer satisfaction across racially and ethnically diverse populations. Our data show that skin evenness and blendability are as critical to product adoption as the prevention of visible residue. Although tailored formulations demonstrated success for mid‐range phototypes (FST III–IV), our results exposed a glaring deficit in the available options for deeper complexions. The clinical rejection of the ‘universal’ variant by FST V–VI subjects confirms that current industry‐standard sunscreens formulations cannot dynamically adapt to the rich chromatic diversity of darker skin and under‐tones. Since aesthetic suitability is an absolute prerequisite for photoprotective compliance, bridging this formulation gap is essential to ensuring that individuals with SOC receive equitable protection options against UV and visible light damage.

## 5 Conclusion

In summary, while the growing popularity of tinted sunscreens continues, the market suffers from limited shade ranges that often exclude darker skin tones, despite claims of “universal” shades or “no white cast”. We hope that our new research provides a deeper understanding of the key aspects that determine successful color matching and product satisfaction in individuals with skin of color. To address these gaps, the industry collectively needs to further develop sophisticated, diverse shade ranges and explore advanced UV filters and non‐iron‐oxide ingredients that provide optimal visible light protection without heavy tinting, while preserving aesthetic acceptability. Clinically, we hope that our results advocate for a more holistic, personalized approach to sunscreen counseling in supporting clinicians and patients navigating formulations tailored to their unique undertones, recognizing that factors like skin evenness and blendability are just as critical to product adoption, ultimately fostering long‐term daily compliance and optimal photoprotection, specifically for patients with darker skin tones.

## Conflicts of Interest

H.D., M.M.Y., T.K.H. are employees of La Roche‐Posay Laboratoire Dermatologique, L'Oreal USA. L.M., A.D., L.C. are employees of QIMA Life Sciences. Z.D.D. is a researcher and consultant for L'Oreal USA. The authors declare no conflicts of interest.

## Data Availability

Research data are not shared.
